# Massive hemoptysis managed by prolonged anticoagulation-free veno-venous extracorporeal membrane oxygenation with which thoracic surgeries were accompanied: a case report

**DOI:** 10.1186/s40981-022-00503-w

**Published:** 2022-02-21

**Authors:** Tomonori Kimura, Tomoe Kobayashi, Fumimasa Kobori, Maria Goto, Mikako Takemitsu, Atsuko Tanaka, Jiro Sato, Shinji Isomine

**Affiliations:** Department of Anesthesiology and Intensive Care Medicine, Funabashi Municipal Medical Center, 1-21-1 Kanasugi, Funabashi, Chiba, 273-8588 Japan

**Keywords:** Massive hemoptysis, Respiratory failure, Anticoagulation-free veno-venous extracorporeal membrane oxygenation, Thoracic surgeries, Endovascular interventions

## Abstract

**Background:**

Massive hemoptysis causing inadequate ventilation results in life-threatening consequences. We present a patient who developed respiratory insufficiency produced by bronchiectatic massive hemoptysis and underwent prolonged anticoagulation-free veno-venous extracorporeal membrane oxygenation (VV-ECMO) during which thoracic surgeries were performed.

**Case presentation:**

A 79-year-old woman suffered massive hemoptysis resulting in respiratory failure during fiberoptic bronchoscopy. Bronchial intubation followed by one lung ventilation failed to ensure adequate oxygenation. Anticoagulation-free VV-ECMO, therefore, was installed immediately. Since conservative hemostatic measures including bronchial arterial embolization were not effective, resection of the culprit lung was performed while on VV-ECMO. Next day an exploratory thoracotomy and intercostal artery embolization were needed for recurrent bleeding. The VV-ECMO was withdrawn after five days of operation.

**Conclusions:**

Massive hemoptysis can be fatal and needs instantaneous and intensive treatments. In our case, long-term anticoagulation-free VV-ECMO during which thoracic surgeries and endovascular interventions were performed provided a favorable outcome.

## Background

Massive hemoptysis is a rare but serious complication of bronchiectasis. As long as pulmonary bleeding remains uncontrolled, it causes deprivation of gas exchange area resulting in lethal outcomes [1, 2]. Insufficient oxygenation through native lungs would claim an installation of artificial lung support [3]. In addition, unless conservative hemostatic measures work such as bronchial tamponade and bronchial arterial embolization (BAE), surgical options including resection of the culprit lesion should be considered [1, 2]. We report a patient who developed respiratory failure due to massive hemoptysis and was successfully treated by prolonged anticoagulation-free veno-venous extracorporeal membrane oxygenation (VV-ECMO) in conjunction with both conservative and surgical hemostatic measures. The report is, to our knowledge, the first to describe the anesthetic and perioperative managements of a patient who suffered persistent hemoptysis-induced respiratory insufficiency surmounted by prolonged anticoagulation-free VV-ECMO.

## Case presentation

**Fig. 1:**
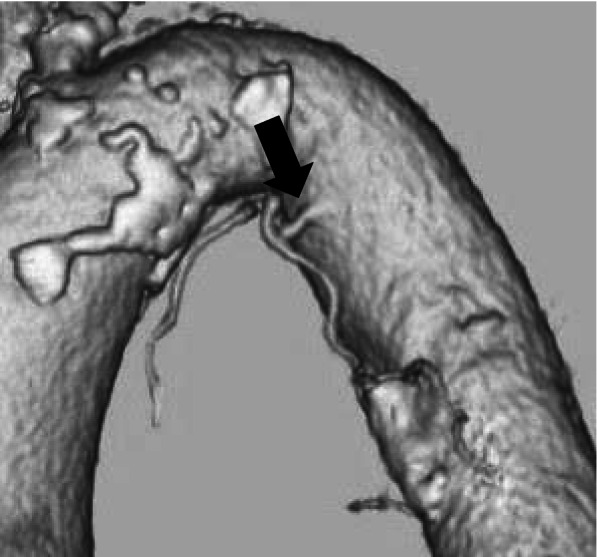
Computed tomography angiography of bronchial arteries. The arrow indicates the common trunk of bronchial arteries which arose from the thoracic aorta

A 79-year-old woman presented with recurrent cough and hemoptysis. A computed tomography (CT) and laboratory studies indicated bronchiectasis in the lower lobe of the left lung associated with allergic bronchopulmonary mycosis. One month later, a sudden and massive hemoptysis prompted an emergency fiberoptic bronchoscopy (FOB), which exhibited substantial bleeding in the left lung and its aspiration into the right lung. A single-lumen endotracheal tube was immediately placed in the right main bronchus to isolate the right lung, followed by mechanical one lung ventilation (OLV). We had to choose a single-lumen tube in the endoscopy room unequipped for emergency airway management. An emergency computed-tomography angiography (CTA) revealed a bulged left bronchial artery, urging BAE. The common trunk of bronchial arteries arose from the thoracic aorta (Fig. 1), complicating selective advancement of an embolization catheter. The BAE failed to achieve satisfactory hemostasis. During BAE, oxygenation worsened down to SpO_2_ 40%. A shift to bilateral mechanical ventilation provided a slight amelioration in blood gas analysis at F_i_O_2_ 0.35; pH 7.37, PaCO_2_, 35.5 mmHg, PaO_2_ 104 mmHg, HCO_3_^−^ 20.2 mmol/l, and BE − 4.5 mmol/l. However, we were afraid that severely decreased lung compliance produced by persistent blood afflux in both lungs would hamper sufficient and protective mechanical ventilation. We, thereby, decided to install VV-ECMO using a poly-2-methoxyethylacrylate (PMEA)-coated circuit (Capiox®, TERUMO, Japan) withholding the use of anticoagulants with the setting of pump speed 1500 rpm, pump flow 2 L/min, O_2_ flow, 2 L/min. The coagulation system examinations following the installation of ECMO were activated partial thromboplastin time (APTT) 40 s and serum fibrinogen 158 mg/dl.

**Fig. 2:**
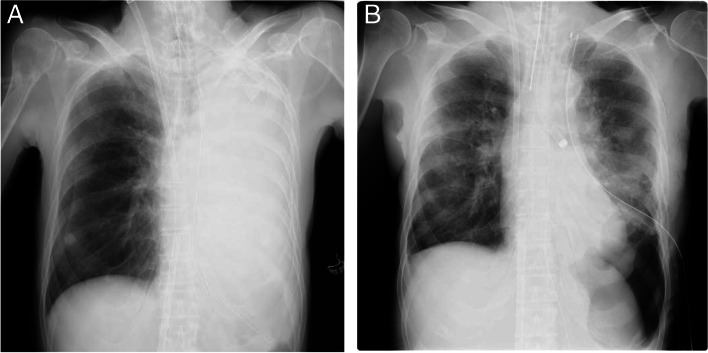
**A** Chest X-ray taken on day 2. It showed diffuse consolidation of the left lung due to massive hemoptysis, indicating whole lung atelectasis. **B** Chest X-ray taken after surgery on day 4. It showed good aeration in the resting left upper lung

Following 2 days, no apparent active bleeding observed let us confine only to performing FOBs for bronchial cleaning, hoping for spontaneous hemostasis. The finding and lowering extracorporeal membrane oxygenation (ECMO) support (F_i_O_2_ 1.0 to 0.5) suggested possible withdrawal from VV-ECMO despite chest X-rays manifesting atelectasis in the whole left lung (Fig. 2A). On day 3, however, an FOB found active rebleeding in the lateral and posterior basal bronchi, where thrombin solution was instilled. The single-lumen tube was replaced by a 35-Fr left-sided double-lumen endobronchial tube through which only the right lung was ventilated and the left lung was kept pressurized at a constant airway pressure 10 cmH_2_O with 100% O_2_, intending astriction. Notwithstanding the efforts, we thought such conservative means were only palliative and a radical surgical measure should be adopted. In the meantime, ECMO weaning trials were carried out in accordance with the Extracorporeal Life Support Organization guideline [4], indicating possible weaning. The ECMO was, however, kept operated at the minimal setting, pump speed 1250 rpm, pump flow 1.5 L/min, O_2_ flow 0.5 L/min, in preparation for surgery-associated worsening of gas exchange and unexpected hemorrhage. Preoperative total amounts of blood products transfused were fresh frozen plasma (FFP) 6 units, packed red cells (PRC) 6 units and platelets 10 units. The preoperative APTT was 42 s and serum fibrinogen 173 mg/dl.

On day 4, resection of the left lower lung lobe was scheduled under inhalational anesthesia with sevoflurane while the patient was on ECMO. We were concerned about unstable depth of intravenous anesthesia produced by abrupt changes in hemodynamics and circulation volume. Depth of anesthesia was closely monitored with the bispectral index (BIS®, Medtronic, USA). The VV-ECMO remained well-controlled during the surgery without major cardiovascular or respiratory events. The surgery achieved considerable hemostasis, with the operation duration 3 h 51 min and intraoperative bleeding volume 863 ml. She was transfused with FFP 8 units, PRC 10 units, and platelets 20 units. Postoperative chest X-ray showed good aeration in the resting left upper lung (Fig. 2B). Bilateral mechanical ventilation presented a marked improvement in gas exchange. However, VV-ECMO still remained operated in the postoperative ICU at the minimal setting since unstable hemodynamics and slowly progressing anemia were sustained.

On day 5, the patient developed a hematoma in the left thoracic wall. An exploratory thoracotomy was performed, achieving hemostasis. Intraoperative bleeding of 2500 ml was compensated by transfusions of FFP 18 units, PRC 12 units and platelets 20 units. Serum fibrinogen was below 100 mg/dl preoperatively but recovered to 132 mg/dl after surgery. For inspection of intravascular emboli formed possibly after prolonged anticoagulation-free ECMO, a postoperative CTA was performed and found, instead, extravasation of contrast medium from intercostal arteries. Transcatheter arterial embolization (TAE) provided a dramatic hemodynamic stability, enabling weaning from VV-ECMO on the same day. Eventually, VV-ECMO was kept operated without anticoagulation for as long as 5 days. On day 6, a CTA detected floating thrombi in the inferior vena cava and bilateral popliteal veins, which required a continuous heparin administration. She was extubated on day 8 and transferred to a general ward on day 9. She was discharge uneventfully from the hospital on day 53.

## Discussion

There are case studies on patients who underwent pulmonary surgery while on VV-ECMO for major airway involvements [5–7]. Preoperative anticipation of the need of temporary interruptions of lung ventilation and gas exchange failure due to lung comorbidities were main reasons for elective and prophylactic use of intraoperative VV-ECMO. In our patient, on the contrary, ECMO was operated continuously beforehand, started at the instance of massive lung bleeding, and carried out even during and after two surgeries.

We needed to balance among prevention of impermissible amounts of bleeding, safe operation of ECMO and assurance of gas exchange via both native lung ventilation and ECMO. We chose anticoagulation-free VV-ECMO, afraid of anticoagulant-induced exacerbation of bleeding.

Albeit a variety of ECMO surface materials circumventing the use of systemic anticoagulants have long been under development [8], no stable techniques have yet been established for long-term anticoagulation-free operation of VV-ECMO. Although the PMEA-coated system we employed is indicated to reduce circuit-related adverse effects such as inflammatory responses and thrombo-coagulopathies, these findings were obtained only by either in vitro studies or short-term uses. In addition, only a few case-based studies reported feasibility of long-term VV-ECMO with low-dose anticoagulant uses [9, 10]. Therefore, we needed to pay considerable attention to coagulation status in the circulation. We monitored blood coagulability solely by serum fibrinogen and APTT accompanied by a careful observation of the status of blood stream in ECMO circuit. After all, fortunately we did not need to exchange the anticoagulation-free circuit for as long as five days. It is, however, undeniable that indiscernible changes occurred in the coagulation-fibrinolysis system which would lead to clotting in the circuit, oxygenator failure, vessel embolism, and hemorrhagic events, as indicated by deep venous thrombosis detected on day 6.

Massive hemoptysis is known to present higher mortality rates with greater amounts of bleeding, reported to be as high as 78% [1]. We, therefore, kept ECMO operated at the minimal setting even when considered unnecessary to secure a transfusion route before bleeding is definitely controlled. Prolonged VV-ECMO in hemorrhagic patients would be a challenge in balancing between thrombotic and bleeding events. Although we adopted an anticoagulation-free operation of ECMO in an attempt to avoid bleeding risk, it would increase the risk of life-threatening thrombo-coagulopathies such as pulmonary artery thromboembolism. Therefore, other choices which have a potential to circumvent both risks concurrently would have to be considered such as intermittent operation of ECMO and use of direct thrombin inhibitors including argatroban [11].

There are considerations regarding the choice of anesthesia while running ECMO. It may be subject to pharmacokinetic instabilities in intravenous anesthetics produced by changes in circulating blood volume due to intraoperative bleeding and extracorporeal circulation, and drug sequestration by ECMO circuit [12]. We, therefore, employed inhalational anesthesia. However, we might need to keep in mind the possibility of unstable delivery of volatile anesthetics in patients with severely reduced pulmonary area for gas exchange as in this case.

The BAE has been considered a first-line therapy for hemoptysis. It is, however, known to be not necessarily successful because of radiographic visualization of non-communicating arteries, perfusion from collaterals, aberrant bronchial arteries, etc. Moreover, embolization of bronchial to spinal artery collaterals would mislead to a serious consequence [1, 2]. We, therefore, decided not to reinstitute BAE after the failure of the first attempt.

In conclusion, considerable experience and expertise in the management of VV-ECMO would enable safe perioperative management of patients with massive pulmonary bleeding resulting in otherwise non-survivable respiratory failure.

## Data Availability

Not applicable.

## Abbreviations

BAE: Bronchial arterial embolization
VV-ECMO: Veno-venous extracorporeal membrane oxygenation
CT: Computed tomography
FOB: Fiberoptic bronchoscopy
OLV: One lung ventilation
CTA: Computed-tomography angiography
PMEA: Poly-2-methoxyethylacrylate
APTT: Activated partial thromboplastin time
ECMO: Extracorporeal membrane oxygenation
FFP: Fresh frozen plasma
PRC: Packed red cells
TAE: Transcatheter arterial embolization
